# Behavioural Effects of the Commonly Used Fish Anaesthetic Tricaine Methanesulfonate (MS-222) on Zebrafish (*Danio rerio*) and Its Relevance for the Acetic Acid Pain Test

**DOI:** 10.1371/journal.pone.0092116

**Published:** 2014-03-21

**Authors:** Janicke Nordgreen, Fernanda M. Tahamtani, Andrew M. Janczak, Tor Einar Horsberg

**Affiliations:** 1 Department of Food Safety and Infection Biology, NMBU School of Veterinary Science, Oslo, Norway; 2 Animal Welfare Research Group, Department of Production Animal Clinical Sciences, NMBU School of Veterinary Science, Oslo, Norway; University of Wurzburg, Germany

## Abstract

The pros and cons of using anaesthesia when handling fish in connection with experiments are debated. A widely adopted practice is to wait thirty minutes after anaesthesia before behavioural observations are initiated, but information about immediate effects of a treatment is then lost. This is pertinent for responses to acute stressors, such as acid injection in the acetic acid pain test. However, omission of anaesthetics in order to obtain data on immediate responses will compromise the welfare of fish and contribute to experimental noise due to stress. We therefore tested the effect of tricaine methanesulfonate on the behaviour of zebrafish. We predicted that tricaine (MS 222) would decrease swimming velocity and that the control fish would show an increased level of anxiety- and stress-related behaviours compared to the tricaine group. Following acclimatization to the test tank, baseline behaviour was recorded before immersion in either tricaine (168 mg l^−1^, treatment group, N = 8) or tank water (control group, N = 7). Latencies to lose equilibrium and to lose response to touch were registered. The fish was then returned to the test tank, and the latency to regain equilibrium was registered in anaesthetized fish. When equilibrium was regained, and at five, thirty and sixty minutes after the fish had been returned to the test tank, behaviour was recorded. The tricaine fish showed the following responses (mean ± sd): latency to lose equilibrium 22.6 s±3.9; latency to lose response to touch 101.9 s±26.8; latency to regain equilibrium 92.0 s±54.4. Contrary to our predictions, neither treatment caused a change in any of the behaviours registered. This indicates that tricaine has no effect on several commonly used behavioural parameters, and that it may be unnecessary to postpone behavioural observations to 30 min after anaesthesia.

## Introduction

General anaesthetics are commonly used in fish research for immobilisation and reduction of pain, discomfort and handling stress [1]–[4] despite the fact that one seldom knows much about how they influence the variables that are studied. Some authors have therefore discussed whether tests such as the acetic acid pain test should be run on unanaesthetised fish in order to avoid possible confounding effects of the anaesthetic [5], [6]. However, omitting anaesthesia may severely impact the welfare of the research animal as well as contributing to noise in the experiment through stress-induced changes in behaviour and physiology [7]. This is particularly relevant for fish compared to terrestrial lab animals as they have to be removed from their native element in order to be handled, rendering them unable to breathe. Even transport using a container filled with water can induce a marked stress-response [7]. Therefore, the use of anaesthetics in fish research is advocated both from ethical and scientific perspectives. In order to use anaesthesia in experimental protocols without confounding anaesthesia with other factors, it is common practice to postpone observations of behaviour to 30 min after fish are removed from the anaesthetic bath [1], [8]–[10]. However, data on induction and recovery times are lacking for common anaesthetics in many species. A recent review on the use of tricaine in fish anaesthesia [4] reports no data on induction and recovery times for zebrafish. Furthermore, to the author's knowledge, no studies have been done on the pharmacokinetics of tricaine in zebrafish. Tricaine is rapidly excreted across the gills, and in a review of fish anaesthetics, recovery time from concentrations in the range of 110 to 220 mg l^−1^ was suggested to be 3–5 min [11]. A recovery time of 6.5 min following anaesthesia with 200 mg l^−1^ tricaine at 20°C was reported for common carp (*Cyprinus carpio*) [12], and Atlantic cod (*Gadus morhua*) kept at 16°C recovered from anaesthesia with 60 mg l^−1^ tricaine in less than 4 minutes [13]. It is therefore possible that the customary 30 min delay may be longer than necessary, at least for some concentrations of -and exposure times to- tricaine. If this is true, the researcher may lose important information about immediate treatment effects by implementing the 30 min delay. For example, unanaesthetised goldfish injected with acetic acid into their cheek show vigorous avoidance responses directly after injection [14]. Had one anaesthetised the fish and postponed observations for the customary time interval, this response would have gone undetected. However, tricaine has local anaesthetic properties [13], [15], [16], and could thus potentially influence the sensitivity of the nociceptors to stimulation in the earlier part of the test. This would be a reason to postpone early behavioural observations even if the fish had recovered from the anaesthetic effect of tricaine. The degree to which tricaine reaches the injection site, and the strength of a local anaesthetic effect when the fish has been submerged in tricaine prior to injection has not been described. In addition to the lack of data on induction and recovery times, there is also a lack of studies testing the effect on behaviour of anaesthesia alone. As a consequence, the question of whether to use anaesthetics or not, and of how long to postpone behavioural observations, may become a matter of personal choice and preference rather than being based on experimental data on zebrafish behaviour. We therefore used EthoVision XT to test the effect of tricaine on the behaviour of zebrafish. We hypothesized that tricaine would have sedative effects that would last beyond the duration of exposure. We therefore predicted tricaine to decrease swimming speed in fish, at least during the first 30 min after anaesthesia. We also hypothesized that the control fish would be more stressed by netting and transfer between test- and treatment tanks, and predicted that this group would show increased levels of anxiety- and stress-related behaviours as a result of handling, and that these effects would not be seen to the same degree in the tricaine group.

## Materials and Methods

### Ethics statement

All experimental work on live animals was approved by the institutional animal care and use committee at the Norwegian School of Veterinary Science under ID number 5750.

### Animals and husbandry

Eighteen male zebrafish (AB/wt, date of fertilisation: 7^th^ of February 2013, weight: mean ± SD; 0.25±0.03 g) were used for this experiment. Two fish from the control group and one fish from the tricaine group had to be excluded due to methodological problems, leaving eight fish in the tricaine group and seven fish in the control group. The fish were purchased from the experimental biomedicine unit at the Norwegian School of Veterinary Science, and brought into our aquarium facility at least one week prior to testing. The move from the breeding facility to our laboratory took less than five minutes, and the fish were carried in a transport tank filled with home-tank water. They were kept singly in aquariums measuring 50×26×32 cm^3^ with white paper between every other tank so that each fish had visual contact with one neighbour. Fish in visual contact are hereafter referred to as a visual pair. Each tank contained five green and five blue glass marbles to increase the complexity of the home environment. The tanks were placed in a semi-closed system with recirculation of system water through mechanical filters, a carbon filter and a UV-sterilizer and with 2% water exchange per day. The fish were fed three times a day, with live brine shrimp (*Artemia*) in the morning and afternoon, and dry food in the middle of the day (9:00, 12:00 and 15:00 H). The light-schedule was 12:12 light:dark with lights being turned on at 08:00. Water temperature was kept at 27–28°C. Water quality was monitored weekly. The experiment was carried out at the Norwegian School of Veterinary Science during October 2013.

### Experimental design, equipment and substances

**Figure 1:**
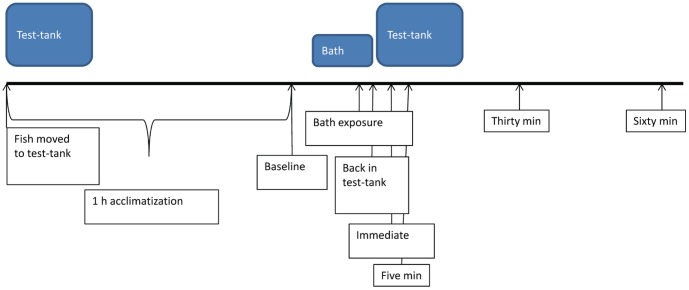
An overview of the experimental design.

**Figure 2:**
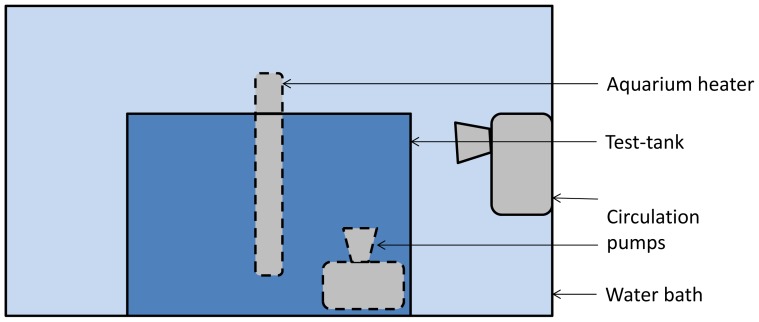
A diagram showing the set-up of the test-tank inside the larger water-tank with an aquarium heater and two circulation pumps placed to ensure a stable temperature. The diagram shows the side of the test-tank facing the camera.

One control and one treatment fish from each visual pair was tested each day (experimental design is shown in Figure 1). Test order was balanced across days. Fish were allocated to treatments in the following manner: after one fish from each visual pair had been distributed randomly to treatment the remaining fish from each pair was allocated to the other treatment. The fish were netted and moved between the home tank and test tank in a net submerged in a water-filled transport container. Later transportation followed the same procedure. The fish were exposed to air when they were lifted between the transport container and the test tank, and the transport container and the treatment bath. At the start of the experiment, each fish was moved to the test tank and left to acclimatize for one hour. The temperature in the test tank was kept between 26.8 and 27.8 °C (mean = 27.4 °C). After acclimatization, baseline behaviour was recorded for ten minutes. At the end of the recording, the fish was transferred to the tricaine bath (tricaine, 168 mg l^−1^; Finquel vet., Western Chemicals Inc., Washington, USA) or to the control bath (tank-water). The tricaine dose was based on the recommendation in the zebrafish book (http://zfin.org/zf_info/zfbook/chapt10.html#wptohtml63). The tricaine stock solution had a concentration of 4 mg ml^−1^ and was buffered to a pH between 7 and 7.5 with Tris buffer (Trizma base, Sigma-Aldrich). Both treatment and control baths contained a total volume of 200 ml. The temperature in the baths was kept between 26.8 and 28.1 °C (mean = 27.5 °C). For the treatment group, latency to lose equilibrium and latency to lose response to touch was registered. The response to touch was tested by gently pressing the tail between two fingers while the fish remained in the bath. When a fish loses response to touch, it is no longer conscious of external stimuli, and can be handled [17]. Upon loss of response to touch, the fish were transported in a container filled with water back to the test tank, and the latency from their return to regaining equilibrium was registered. The control fish were kept in the control bath for two minutes and thirty seconds, and were then put back into the test tank. Behaviour was recorded from when the treatment fish regained equilibrium or the control fish were put back into the test tank starting immediately (‘immediate’), and at five (‘5-min’), thirty (‘30-min’) and sixty minutes (‘60-min’) after the fish were put back into the test tank. All recordings, except for immediate recordings, lasted for ten minutes. The immediate recording lasted until the start of the 5-min recording. The glass test tank was 21.5 cm×13.5 cm×25 cm (length x width x height), and covered on three of the inside walls and on the floor with white plastic giving a test-arena of 20 cm×8 cm×17.4 cm. The fish were filmed through one of the long sides of the tank with a Panasonic color CCTV camera (WV-CP500/G). The subdivision of the test tank into front (test-arena) and back compartments allowed us to keep thermometers in the back part of the tank during testing without having them disturb the fish or make tracking of the fish difficult. The test tank was submerged in a larger tank, containing an aquarium heater and two circulation water pumps to ensure a stable temperature (Figure 2). The fish were tracked using the EthoVision XT 9 software (Noldus, Wageningen, The Netherlands). The dependent variables and rationale for choosing them are described in Table 1. Freezing was scored manually using direct observation of focal fish in EthoVision.

**Table 1:** The behaviours registered and our rationale for choosing them.

Behaviour	Unit/definition	Background for choice of behaviour
Swimming velocity	cm	A measure of sedation.
Total time spent in lower third of the tank	s	A measure of anxiety in the novel tank diving test [28]–[30]
Frequency of zone transition between the lower third and the rest of the tank		A measure of anxiety in the novel tank diving test [28], [30]
Latency to leave the lower third of the tank	s	A measure of anxiety in the novel tank diving test [14], [16]
Cumulative duration of time spent in the central part of the tank	s	Thigmotaxis is used as a measure of anxiety in zebrafish [29], [31], [32].
Freezing	Being stationary with only minor body movements	This behaviour was divided into two categories: freezing with or without body in contact with the bottom of the tank. Freezing can indicate fear or anxiety [29], [32], [33]. Freezing in contact with the tank bottom may indicate sedation.

### Statistical analysis

The data were analysed using a repeated measures ANOVA with fish as a random factor and time and treatment as fixed factors. Fish were nested in treatment, and the time by treatment interaction was included in the model. When data could not be transformed to fulfil the requirements of GLM (normality of error, homogeneity of variance and linearity), non-parametric tests were used. For comparison of non-parametric variables that could not be adequately transformed the Wilcoxon matched pairs signed rank test was used to compare values within treatment group and the Wilcoxon test for unpaired data was used to compare values between treatment groups. Latency data were also analysed as described above. This is because no fish had to be assigned a maximum latency, ie: all fish left the lower third of the tank before the recording was ended, and we therefore chose not to do a survival analysis on the results, a method which is often recommended for latency data when a cutoff or maximum value has to be assigned to non-responding subjects [18]. Variables that fulfilled the assumptions of parametric statistics are presented as mean ± sd, whereas variables that did not are presented as median with the 25% quartile (Q1) and 75% quartile (Q3). We compared the baseline measures with measures from later time-points within each treatment group, and we also compared the two different treatment groups for each time-point. All tests were two-tailed and the critical p-value was 0.05. As observation periods were not of equal duration, data on durations of behaviour were transformed to percentage of the duration of the observation period in order to allow comparison of behaviour recorded in different observation periods. All statistical analysis was performed using JMP version 10.0.0.

## Results

The mean latency to lose equilibrium and response to touch and to regain equilibrium for the tricaine -treated fish are shown in table 2. The mean ± sd or median with the 25% quartile (Q1) and 75% quartile (Q3) of each of the variables measured are presented in table 3. Freezing in the water column was observed in less than 50% of the fish. A visual assessment of the data showed that freezing occurred equally rarely in both treatment groups, and the results were not analysed further. Freezing on the bottom was never observed.

**Table 2:** Mean latency from being placed in the anaesthetic bath to loss of equilibrium, from being placed in the anaesthetic bath to loss of response to touch, and from return to the test tank to regaining equilibrium in tricaine -treated fish.

Variable	Mean (s)	Standard deviation
Latency to lose equilibrium	22.6	3.9
Latency to lose response to touch	101.9	26.8
Latency to regain equilibrium	92.0	54.4

**Table 3:** Mean ± sd or median with the 25% quartile (Q1) and 75% quartile (Q3) for each of the behavioural variables.

Variable	Time	Tricaine (n = 8)	Control (n = 7)
Mean velocity (cm/s)	Baseline	4.0±0.9	3.3±1.5
	Immediate	3.0±0.6	3.7±1.2
	5-min	3.0±0.9	3.0±1.2
	30-min	3.0±1.0	3.5±2.1
	60-min	3.6±1.5	3.0±1.0
Time spent in lower third of the tank relative to duration of trial (percentage of duration of observation period)	Baseline	51.6 (26.4–68.0)	59.7 (26.1–80.7)
	Immediate	56.0 (41.9–71.2)	53.0 (43.9–95.9)
	5-min	60.3 (21.4–87.1)	60.0 (37.8–89.3)
	30-min	47.1 (26.4–92.2)	73.3 (11.1–89.9)
	60-min	66.0 (24.2–93.6)	80.1 (46.7–94.7)
Frequency of zone transitions between the lower third and the rest of the tank (per min)	Baseline	4.3±2.9	3.8±3.0
	Immediate	2.6±0.7	2.1±1.5
	5-min	2.3±1.5	2.1±1.6
	30-min	2.5±2.1	1.7±1.4
	60-min	2.6±2.9	1.2±0.6
Latency to leave the lower third of the tank (s)	Baseline	17.8 (10.3–29.0)	7.9 (4.8–59.0)
	Immediate	22.2 (6.1–68.4)	19.2 (9.3–215.8)
	5-min	12.1 (8.2–18.7)	8.6 (3.9–64.5)
	30-min	8.8 (1.7–36.7)	24.0 (14.9–36.8)
	60-min	5.2 (2.5–34.5)	16.8 (14.8–29.0)
Cumulative duration in the central part of the tank relative to the total duration of the trial (percentage of duration of observation period)	Baseline	44.2±23.6	36.1±24.7
	Immediate	46.3±15.9	30.2±23.1
	5-min	39.6±23.5	38.3±28.2
	30-min	32.7±18.0	23.0±18.3
	60-min	32.5±17.3	20.5±9.8

The mean velocity was neither affected by treatment (F_1, 13_ = 0.0045; p = 0.95), time (F_4, 52_ = 0.56; p = 0.69) or the treatment by time interaction (F_4, 52_ = 1.11; p = 0.36). Time spent in the bottom third of the tank differed from normality and was therefore analysed with the Wilcoxon matched pairs signed rank test. There were no differences between behaviour during baseline and later time-periods within any group (control: baseline vs immediate: df = 6, S = 7, p = 0.30; baseline vs 5-min: df = 6, S = 5, p = 0.47; baseline vs 30-min: df = 6, S = 3, p = 0.69; baseline vs 60-min: df = 6, S = 4, p = 0.58. MS-222: baseline vs immediate: df = 7, S = 3, p = 0.74; baseline vs 5-min: df = 7, S = −1, p = 0.95; baseline vs 30-min: df = 7, S = 4, p = 0.64; baseline vs 60-min: df = 7, S = 5, p = 0.55). The treatment and control group did not differ at any time-point (Wilcoxon test for non-paired data, 2-sample test, normal approximation: baseline:S = 57, Z = 0.058, p = 0.95; immediate: S = 59, Z = 0.29, p = 0.77; five min: S = 59, Z = 0.29, p = 0.77; thirty min: S = 57, Z = 0.058, p = 0.95; sixty min: S = 61, Z = 0.52, p = 0.60).The variable zone transition was root-transformed. There was a tendency towards an effect of time F _4, 52_ = 2.16, p<0.086 with fewer zone transitions at 60 minutes than at baseline (post-hoc Tukey, p = 0.071). Latency to leave the bottom zone could not be transformed to fulfil the assumptions of ANOVA, and was analysed using the Wilcoxon matched pairs signed rank test. There were no differences between baseline and post-treatment time-points within any of the two treatment groups, and the treatment groups did not differ at any time-point (Within-group comparisons: control: baseline vs immediate: df = 6, S = 6, p = 0.38; baseline vs 5-min: df = 6, S = −1, p = 0.94; baseline vs 30-min: df = 6, S = 6, p = 0.38; baseline vs 60-min: df = 6, S = 3, p = 0.69. MS-222: baseline vs immediate: df = 7, S = -2, p = 0.84; baseline vs 5-min: df = 7, S = −5, p = 0.55; baseline vs 30-min: df = 7, S = −3, p = 0.74; baseline vs 60-min: df = 7, S = −11, p = 0.15. Between-group comparisons: baseline: S = 43, Z = −1.45 p = 0.15; immediate: S = 59, Z = 0.29, p = 0.77; five min: S = 55, Z = −0.058, p = 0.95; thirty min: S = 68, Z = 1.33, p = 0.18; sixty min: S = 70, Z = 1.56, p = 0.12).

Time spent in the centre zone relative to the duration of the trial was root-transformed. The ANOVA indicated a significant effect of time: F _4,52_ = 2.58; p<0.048. However, the post-hoc Tukey test did not indicate any significant differences or any tendencies (with p<0.1) between observation periods.

## Discussion

Neither anaesthesia to a level of depth where the fish did not respond to touch, nor being moved between tanks affected the behaviour of zebrafish in this study. The lack of an effect on locomotion lasting beyond the actual exposure to the anaesthetic is surprising and contrary to our prediction, which was based on the commonly used protocol of waiting 30 min between anaesthesia and behavioural observation [1], [8]–[10], [19]. The lack of an effect of tricaine on behaviour has two important implications. Firstly, there is no reason to omit anaesthesia in zebrafish in order to avoid confounding effects on the behaviours recorded in the present study. Secondly, when anaesthesia is applied, one could potentially start registering effects of the treatment as soon as the fish regains equilibrium, thus allowing observation of acute responses. This is very relevant for the commonly used acetic acid injection pain test in fish. The acetic acid may have damaging effects on tissue [14] including nociceptors [20], and the immediate response may therefore be different from the response that can be measured after 30 min or later. In addition, if immediate responses to the acid injection can be measured, the duration of the test could be decreased. This would be a refinement of the test as the fish would experience pain for a shorter period of time. However, there are two factors to consider before the current results can be applied to the acetic acid test in practice: firstly, in the acetic acid test the fish are exposed to air during injection, and sometimes during weighing as well. The tricaine group in the current experiment was not exposed to air. Air exposure could lead to hypoxia, which could influence behaviour in a way that this study cannot predict. Importantly, omitting anaesthesia would not alleviate this problem, but rather exacerbate it due to increased demand for oxygen in unanaesthetised fish. An additional factor to take into consideration when discussing the use of tricaine in the acetic acid test is the local anaesthetic properties of this substance [15]. Tricaine works by blocking voltage-sensitive sodium channels (ibid; [21]), and it significantly decreases the activity in the lateral line nerve of oyster toadfish (*Opsanus tau*) [16] and decreases the sensitivity to depolarizing current in supramedullary/dorsal neurons in the cunner (*Tautogolabrus adspersus*) [22]. Thus the response to acid may be weak during the first 30 minutes after anaesthesia, and this would make early observations of behaviour of little relevance. The strength of the local anaesthetic effect is influenced by access of tricaine to the injection site, but this has to our knowledge not been studied previously. By observing the fish response to acetic acid during the first 30 minutes after anaesthesia and acetic acid injection, the impact of the local anaesthetic effects of tricaine on the pain response could be tested. This question should be clarified before one makes recommendations regarding when to start behavioural observations in the acetic acid test.

There are some restraints on generalization of the present results. Firstly, the anaesthetic concentration, size of fish, strain of fish, temperature and pH are some of the factors that may influence both induction and recovery times (reviewed by [11], [17]). The concentration of tricaine used in the present study (168 mg l^−1^) is high. Correia et al. (2011)[10] anaesthetized zebrafish in 50 mg l^−1^ of tricaine before injecting them with acetic acid. An LC_50_ of 170 mg l^−1^ was reported by Sanches-Vazquez et al. (2011)[23], but this was after 15 min of exposure. In the same paper, the minimum effective concentration was reported to be 50 mg l^−1^. A 15 min exposure to 60 mg l^−1^ led to an increased swimming activity in the top of the tank for 4 minutes, and then reduced activity for the whole 30 min recording period (*ibid*). This is in contrast to the current findings, but illustrates the importance of exposure duration and anaesthetic concentration. To the authors' knowledge, the effect of zebrafish strain on the response to tricaine has not been tested. Secondly, the behaviours that were recorded in the current experiment are relatively simple. It is possible that more cognitively demanding tasks may be influenced by anaesthesia to a larger degree than swimming and position in the tank. Juvenile salmonids that had been trained in an orienting task lost their learnt orientation towards the direction of food for several days after anaesthesia in tricaine [24]. Lastly, the acetic acid test is often carried out in the home-tank of the fish (e.g. [2], [9]). Testing the fish in a novel test-tank as we did in the current experiment could induce stress. The frequency and duration of behaviours reported in the current study may be different from those that would have been observed in the home-tanks.

The lack of a behavioural change in the control fish was also contrary to our predictions which were based on the hypothesis that handling causes stress. Previous studies report that netting and transfer between tanks can cause severe stress responses ([7]). The lack of handling effects in the present study could potentially be explained by a ceiling effect if transfer to the test tank in itself caused maximal stress responses. However, the behaviour of fish during baseline makes this explanation unlikely. We observed little erratic swimming. Most fish swam at medium speed with short stops and turns. Their behaviour in the test tank was thus very similar to their behaviour in the home-tanks. Another possible explanation of the stability of behaviour in the control fish is that the handling was mild (with netting above the water level kept to an absolute minimum), and that the fish were well-habituated to their surroundings. Furthermore, the fish had not been exposed to a long stressful transportation to our aquarium facilities. The transportation between the breeding facility and our aquarium took less than 5 minutes. We also avoided the common procedure of fasting fish prior to observation as we believe that omission of anticipated food is a potent stressor. Fish can anticipate their next meal when they are fed at regular intervals [25], and show signs of frustration when an anticipated meal is withheld [26].

While this experiment was carried out, a paper was published showing that tricaine is aversive to zebrafish [27]. As tricaine is the most widely used anaesthetic for zebrafish, and will probably continue to be used in the future, we believe that our results are important for the development of ‘best practice’ in experimental anaesthesia protocols. However, the findings of Readman and colleagues [21] highlight the need for further studies of induction and recovery latencies as well as effects on behaviour for several anaesthetics in zebrafish, so that the pros and cons of each substance can be properly compared.
